# Measuring the Meltdown: Drivers of Global Amphibian Extinction and Decline

**DOI:** 10.1371/journal.pone.0001636

**Published:** 2008-02-20

**Authors:** Navjot S. Sodhi, David Bickford, Arvin C. Diesmos, Tien Ming Lee, Lian Pin Koh, Barry W. Brook, Cagan H. Sekercioglu, Corey J. A. Bradshaw

**Affiliations:** 1 Department of Biological Sciences, National University of Singapore, Singapore, Singapore; 2 Herpetology Section, Zoology Division, National Museum of the Philippines, Manila, Philippines; 3 Ecology, Behavior and Evolution Section, Division of Biological Sciences, University of California San Diego, La Jolla, California, United States of America; 4 Department of Ecology and Evolutionary Biology, Princeton University, Princeton, New Jersey, United States of America; 5 Research Institute for Climate Change and Sustainability, School of Earth and Environmental Sciences, University of Adelaide, Adelaide, South Australia, Australia; 6 Department of Biological Sciences, Stanford University, Stanford, California, United States of America; 7 School for Environmental Research, Institute of Advanced Studies, Charles Darwin University, Darwin, Northern Territory, Australia; University of Sheffield, United Kingdom

## Abstract

Habitat loss, climate change, over-exploitation, disease and other factors have been hypothesised in the global decline of amphibian biodiversity. However, the relative importance of and synergies among different drivers are still poorly understood. We present the largest global analysis of roughly 45% of known amphibians (2,583 species) to quantify the influences of life history, climate, human density and habitat loss on declines and extinction risk. Multi-model Bayesian inference reveals that large amphibian species with small geographic range and pronounced seasonality in temperature and precipitation are most likely to be Red-Listed by IUCN. Elevated habitat loss and human densities are also correlated with high threat risk. Range size, habitat loss and more extreme seasonality in precipitation contributed to decline risk in the 2,454 species that declined between 1980 and 2004, compared to species that were stable (*n* = 1,545) or had increased (*n* = 28). These empirical results show that amphibian species with restricted ranges should be urgently targeted for conservation.

## Introduction

Amphibians epitomise the modern biodiversity crisis, having exhibited major population declines, disease susceptibility, morphological deformities, and well-publicized recent extinctions [1], [2]. The recent global amphibian assessment [2] showed that 32% of the world's amphibian species are unequivocally threatened with extinction, with another 22.5% too poorly studied to warrant exclusion from or addition to this growing list. Over 160 amphibian species are thought to have become extinct in recent decades, and at least 43% of all described species are currently experiencing population declines [2]. Thus, amphibian species represent an especially sensitive bellwether to habitat and climate change [3]–[6]. Although various threats to amphibians (e.g., global warming, habitat loss, disease vulnerability to chytrid fungus, and pollution) have been identified [4]–[7], large-scale analyses of extinction risk in amphibians have been few [8]. This situation impedes tangible conservation and identification of threatened amphibians because localised or small-sample studies restrict inference over the entire Class.

In general, large body size and small range are the most common threat risk correlates identified for almost all organisms examined to date. With decreasing range, a species' populations are thought to be more susceptible to localised stochastic events [9], and larger body sizes generally correlate with slower life history traits, thus impeding recovery potential after population crashes [10]. These traits may also be important in explaining population decline and extinction risk in frogs [8], [11], [12]. However, previous studies have been limited in scope, either due to a small number of species examined (the largest sample thus far represents <10% of all amphibian species–[8]) or restricted geographic area [e.g., refs. 12], [13]. While we acknowledge that local drivers can be important, testing for major global drivers helps put local effects into a general context where they can be more easily evaluated, measured, and probably controlled. Using an extensive database describing ecological, life history and environmental attributes of approximately 45–60% of all known amphibian species (3,366 species; some species were excluded from analysis because of incomplete data; see Results), we determined which traits were most associated with threat and decline risks (see Materials and Methods).

Our data represent nearly an order of magnitude more species than any previous study and have representatives from all three amphibian orders (Anura [frogs and toads], Caudata [salamanders] and Gymnophiona [caecilians]), something no other study has yet achieved (see Table S1). Our analyses are also based on the multi-model inferential paradigm that differs from Neyman-Pearson hypothesis testing in that the former achieves stronger inference in cases of multivariate causality [14]–[16]. This approach has been used successfully for exploring determinants of extinction and threat risk in other taxa [e.g., 17], [18], and we apply it here to determine the relative strengths of evidence for different aspects of amphibian life history, geography, and other variables to explain threat risk. Additionally, because drivers of population decline are often decoupled from stochastic factors that cause eventual extinction [17], we determined whether declining amphibian species (between 1980 and 2004) were affected by habitat loss, climate, life-history and ecology.

We examined the following specific, but related questions: (1) Does habitat loss (and human density as a surrogate measure of habitat loss) affect amphibian endangerment and decline? (2) Do temperature and precipitation (as proxies of climate change and potential disease susceptibility) affect amphibian endangerment and decline? (3) What aspects of ecology and life history (e.g., range size, body size and reproductive mode) are most important in determining amphibian endangerment and decline? (4) Do different processes affect endangerment and decline? For example, is there evidence for interactive effects between drivers on the risk of threat and population decline?

## Results and Discussion

To avoid circularity, we excluded those species categorized on the basis of geographic range (IUCN “B” criteria) and used the remaining 2,494–3,052 amphibian species (depending on the specific analysis–see below) for our analysis. Threat categories were combined into “threatened” (*Critically Endangered, Endangered, Vulnerable*, *Near Threatened*) and “non-threatened” (*Least Concern*) categories. Despite a relatively large dataset (435 threatened versus 2,059 non-threatened species), *geographic range* alone still explained nearly half of deviance in threat risk (percentage deviance explained [%DE] = 45%), but there was little evidence for a nonlinear (quadratic) effect of range on threat risk as reported by Cooper et al. [8] (Table 1a). According to the dimension-consistent Bayesian Information Criterion (BIC) weights (*w*BIC), a method for inferring strength of evidence of a statistical model appropriate for large samples with tapering effects [14], a correlative model including only *geographic range* and *body size* had majority support (*w*BIC = 0.975). The inclusion of all interactions in the fully saturated models raised %DE by only ∼2%. The body size-only model accounted for only about 1% of the deviance in threat risk. Spatial autocorrelation did modify model rankings and goodness-of-fit slightly (Tables 1 and S6, S7 and S8); even though the BIC evidence ratio indicated that the top-ranked model without spatial autocorrelation was nearly 14 times better supported than when it included spatial autocorrelation. In general, threat risk decreases linearly with an increase in the log of *geographic range* or *body size*. Other ecological and life history attributes received little relative support, with only weak evidence for life history *habit* (terrestrial, aquatic, terrestrial-aquatic, or arboreal lifestyles) and *reproductive cycle* terms. The highest-ranked GLMM based on the reduced dataset, when re-parameterized as a simple generalized linear model (GLM), revealed a %DE of 44.69%. This demonstrates only a small effect of phylogeny on explained variance in threat risk (although model ranking changes–Supplementary Tables S6, S7 and S8).

**Table 1:** Correlates of amphibian threat risk.

Model	*k*	*LL*	ΔBIC	*w*BIC	%DE	Δ%DE
(a) Ecology/life-history						
BS+RG	8	−580.785	0.000	0.975	46.11	
BS+RG+HB+RC	12	−575.106	8.972	0.011	46.63	
RG+RG^2^	8	−585.825	10.188	0.006	46.64	
BS+RG+HB+FT	12	−575.858	10.519	0.005	46.56	
BS+RG+HB+PC	12	−577.347	13.627	0.001	46.43	
(b) Environmental context						
BS+RG+TM+PV	10	−572.935	0.000	0.354	48.53	2.42
BS+RG+TM+PM+PV	11	−570.693	0.564	0.267	48.73	2.62
BS+RG+TM+TV+PV	11	−570.907	0.980	0.217	48.71	2.60
BS+RG+TM+PV+HL	11	−572.290	3.787	0.053	48.59	2.48
BS+RG+TM+PM+PV+HL	12	−569.931	4.102	0.046	48.80	2.69

The five most parsimonious generalized linear mixed-effects models investigating (a) life history correlates of threat risk (*n* = 2,494) and (b) environmental context, after accounting for effects of range and body size (*n* = 2,584). Models include nested (hierarchical) taxonomic (Order/Family) random intercepts and geographic distance random slopes to account for spatial autocorrelation. Models were ranked according to the Bayesian Information Criterion (BIC). For ecology/life history models, the five most highly BIC-ranked models accounted for >99 % of the posterior model weight (*w*BIC) of the total of 40 models considered. For environmental context, model weights were more evenly distributed among the 5 most highly ranked of the 75 models considered. Terms shown are RG = *range* (km^2^), BS = *body size*, HB = *habit*, RC = *reproductive cycle*, PC = *presence/absence of parental care*, and FT = *fertilization type*, TM = *mean temperature*, PV = *precipitation range*, PM = *mean precipitation*, TV = *temperature range*, HL = *% habitat lost*, HD = *human density* (people/km^2^) Also shown are number of parameters (*k*), maximum log-likelihood (*LL*), difference in BIC for each model from the most parsimonious model (ΔBIC) model weight (*w*BIC), percent deviance explained (%DE) in the response variable (threat probability) by the model under consideration, and the difference between the %DE for the current environmental context model and the base ∼BS+RG model (Δ%DE).

**Figure 1:**
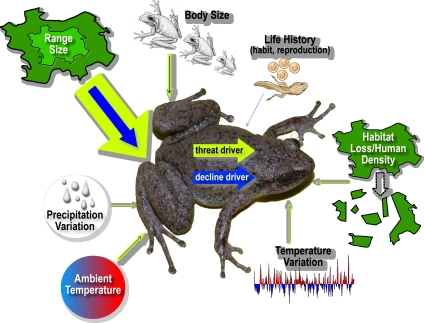
Major variables affecting amphibian species threat (yellow arrows) and decline (blue arrows) risk. Arrow width corresponds to amount of threat or decline risk (approximately related to the per cent deviance explained) described by each attribute (Tables 1 and S5–S6). The major determinant of both threat (IUCN Red-Listed) and decline risk is range size (stronger effect for threat risk), followed by body size (allometry). Certain life history characteristics (life habit, reproductive cycle and mode) also weakly affect decline risk. Environmental conditions such as mean ambient temperature, temperature seasonality, precipitation seasonality, habitat loss and human density also explain a small amount of variation in both threat and decline risk.

We also identified environmental determinants (local context) of threat risk, after controlling for the conditional life history traits of *geographic range* and *body size* (Table 1b). There was some support for weak effects of *mean annual temperature*, *annual temperature seasonality* and *annual precipitation seasonality* (the two top-ranked models accounted for 0.354 and 0.267 of the *w*BIC, respectively; Table 1b). Threat risk increased with more pronounced seasonality in *temperature*, *precipitation*, *habitat loss* and *human density*, but declined with increasing ambient *temperature* (Fig. 1). Although not completely intuitive, these results agree broadly with known environmental and historical constraints on amphibian distributions [19] and suggest that multiple variables may threaten amphibians. Thus there is an urgency with which amphibian restoration efforts must target regions of high amphibian threat risk, given that anthropogenic climate change is known to exacerbate amphibian extinction trends [1].

**Figure 2:**
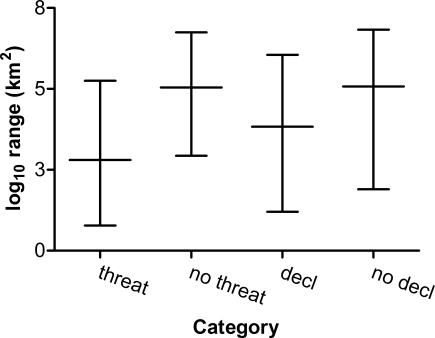
Median geographic range sizes for various amphibian threat and decline categories. Median (±95% confidence limits) log-transformed geographic range sizes for Red-Listed (threatened) versus non-threatened species, and for declining (assessed between 1980 and 2004) and non-declining (stable or increasing) species.

Species of amphibians with small geographic ranges tend to have more habitat specificity [11], which makes them vulnerable to habitat alterations. On the other hand, widespread species tended to be more general in their habitat preferences with the widest diversity of breeding sites. Further, amphibians with small ranges may have low abundance and reproductive success, making them particularly vulnerable [8], [20] (Fig. 2).

**Figure 3:**
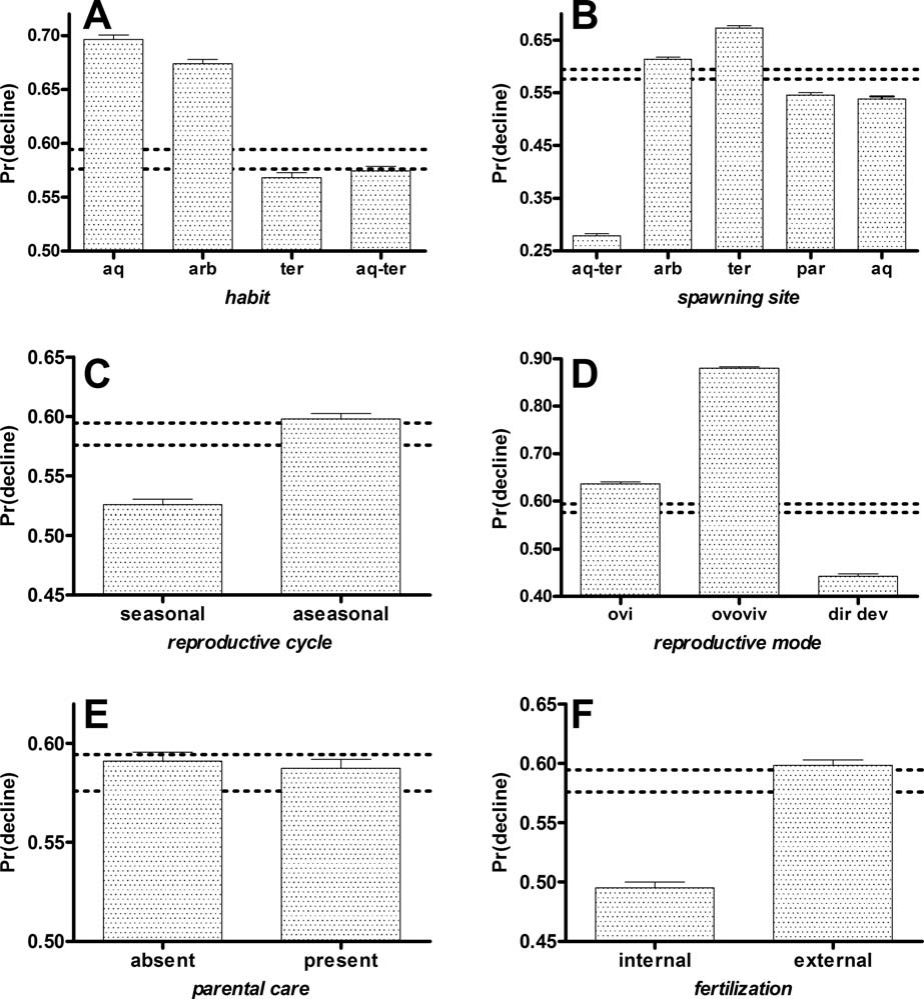
Predicted probabilities of population decline for the life history terms *habit*, *spawning site*, *reproductive cycle*, *reproductive mode*, presence/absence of *parental care* and *fertilization* type (derived from the nine-term model BS+RG+RG^2^+HB+SS+RC+RM+PC+FT based on the BIC-selected top-ranked model; see Table 2). The observed extinction probability 95% confidence interval (dotted horizontal lines) was determined by a 10,000 iteration bootstrap of the probabilities predicted by the above model over 3,052 species. Changes to extinction probability relative to each term level were calculated by adjusting the original dataset so that all species were given the same value for that level (each level value in turn), keeping all other terms in the model as in the original dataset. Error bars represent the 10,000 iteration bootstrapped upper 95% confidence limits. aq = aquatic, arb = arboreal/phytotelms, ter = terrestrial, aq-ter = aquatic & terrestrial, ovi = oviparious, ovoviv = ovoviviparous, dir dev = direct development. See text and Supplementary Table S3 for a description of variables.

Drivers of population decline are often decoupled from stochastic factors that can cause eventual extinction [17], [21]. To distinguish these different processes, we also collected data from 4,027 species with known population trend data from 1980 and 2004 to determine if the same set of ecological, life history and environmental drivers that explained threat, also explained the probability of decline (2,454 declining, 1,545 stable and 28 increasing species). Generally agreeing with the IUCN threat status results, small *geographic range* and large *body size* were still correlated with a higher likelihood of population decline (Table 2; Fig. 1), but there was also evidence for a nonlinear (quadratic) effect of range (Table 2). Further, despite using Bayesian inference to identify the most important drivers of correlations, there were important additional tapering effects not identified in the threat-risk phase: *habit*, *spawning site*, *reproductive cycle*, *reproductive mode*, *parental care* and *fertilization*; these accounted for an additional ∼2.3% of deviance in decline risk above the *body size* and nonlinear *range* model (Table 2a). Aquatic and arboreal species, species with specific spawning requirements, aseasonal breeders, ovoviviparous species and species with external fertilization all appear to have higher risks of declining (Fig. 3).

**Table 2:** Correlates of amphibian decline risk.

Model	*k*	*LL*	ΔBIC	*w*BIC	%DE	Δ%DE
(a) Ecology/life-history						
BS+RG+RG^2^+HB+SS+RC+RS+PC+FT	21	−1598.536	0.000	0.951	17.01	
RG+RG^2^	8	−1642.173	6.314	0.040	14.75	
BS+RG+RG^2^	9	−1640.552	9.346	0.009	14.83	
BS+RG+HB+RC+RS	14	−1644.806	49.419	<0.001	14.61	
BS+RG+HB+RC	12	−1654.167	55.633	<0.001	14.13	
(b) Environmental context						
lhb…+TM+PV+HL	24	−1525.251	0.000	0.934	20.26	3.25
lhb…+TM+TV+PV+HL	25	−1525.143	5.954	0.048	20.26	3.25
lhb…+TM+PM+PV+HL	25	−1526.291	8.083	0.016	20.20	3.19
lhb…+PM+PV+HL	24	−1531.849	13.502	0.001	19.91	2.90
lhb…+TV+PV+HL+HD	25	−1530.015	15.776	<0.001	20.01	3.00

The five most parsimonious generalized linear mixed-effects models investigating (a) life history correlates of decline risk (*n* = 3,045) and (b) environmental context, after accounting for effects of life history correlates (top-ranked ecology/life-history model denoted as ‘lhb’–life-history base) (*n* = 3,121). Models include nested (hierarchical) taxonomic (Order/Family) random intercepts and geographic distance random slopes to account for spatial autocorrelation. Models were ranked according to the Bayesian Information Criterion (BIC). For ecology/life history models, the five most highly BIC-ranked models accounted for >99 % of the posterior model weight (*w*BIC) of the total of 40 models considered. For environmental context, model weights were more evenly distributed among the 5 most highly ranked of the 75 models considered. Terms shown are RG = *range* (km^2^), BS = *body size*, HB = *habit*, RC = *reproductive cycle*, RS = *reproductive strategy*, PC = *presence/absence of parental care*, SS = *spawning site* and FT = *fertilization type*, TM = *mean temperature*, PR = *precipitation range*, PM = *mean precipitation*, TR = *temperature range*, HL = *% habitat lost*, HD = *human density* (people/km^2^) Also shown are number of parameters (*k*), maximum log-likelihood (*LL*), difference in BIC for each model from the most parsimonious model (ΔBIC), model weight (*w*BIC), percent deviance explained (%DE) in the response variable (decline probability) by the model under consideration, and the difference between the %DE for the current environmental context model and the life history base (lhb) model (Δ%DE).

These life history traits (*body size*, *range*, *spawning site*, *reproductive cycle*, *reproductive mode*, *parental care* and *fertilization* type) attributes were set as control variables in the environmental analysis. We found evidence for additional effects of *mean annual temperature*, *annual temperature seasonality*, *annual precipitation seasonality*, *human density* and *proportional habitat loss* on decline risk (Table 2b), although effects were weak (change in %DE between life history control model and best-supported models = 2.9 to 3.3%; Table 2b). Risk of decline decreased under higher ambient *temperature* and increased with greater *precipitation seasonality* and *habitat loss*. Further, and in contrast to the threat status results, decline risk decreased mainly with lower *temperatures*, higher *precipitation seasonality* and increased *habitat loss* (Table 2b). These differences underscore important distinctions between threat status (which is potentially conflated with natural rarity) and decline (Fig. 1).

Studies on birds and mammals have determined that range-restricted and large-bodied species are generally more vulnerable to extinction than their widespread and smaller counterparts [21], [22]. Such species are potentially good indicators of the onset of environmental change, being relatively more sensitive to abnormal climate patterns and habitat loss [23], [24]. Our results both corroborate previous restricted-scale or low-sample studies [e.g., 8], [12], [13], [25], but also deliver new insights to the relative importance and potential synergies of different drivers on both amphibian endangerment and decline risk (e.g., that body size, reproductive characteristics, and most importantly, climate seasonality modify amphibian threat risk). Not only do most studies show that geographic area is one of the most important drivers of extinction risk, our data reveal that it is *the* most important by far relative to all other potential drivers, even though there are a host of other potential drivers modifying the probability weakly. Further, we found no evidence for interactions among drivers; however, it would be interesting to see if this trend holds with even large samples sizes. Another important result of our study is that different datasets, statistical approaches and regional assessments generally agree on important drivers of extinction. Our challenge now is to implement these findings into sound conservation approaches, specifically by targeting range-restricted species.

### Conclusions

We found statistical support for models incorporating the effects of climate seasonality, although its influence on extinction risk relative to range and body size is weak. Our results also highlight the contribution of habitat degradation and human density to amphibian extinction and decline risk. Although threatened and declining amphibian species are constrained by many of the same conditions (e.g., precipitation seasonality), the two indices describe subtly different components of the pathway to extinction [17], [21]. The reasons for population declines that can push a species towards a higher risk of extinction can be a complex function of many factors acting simultaneously [26]. As such, analyses aiming to determine the relative importance potential drivers require large samples and broad geographic coverage to make inference across entire taxa. Our findings that amphibians are more susceptible to decline when they have small geographic ranges and large body sizes are not new; however, our discovery that extrinsic forces increase the susceptibility of high-risk species validates the hypothesis that global warming and the increased climatic variability this entails, spell a particularly dire future for amphibians. Evidence is mounting that both direct (e.g., habitat destruction) and indirect (e.g., climate change) factors now severely threaten amphibian biodiversity [1], [5], [6]. Our study confirms that areas containing high number of restricted range amphibians should have conservation priority. Although efforts such as captive breeding [27] might help to buffer some declining populations in the short term, such interventions cannot substitute for habitat protection and restoration. The synergies between ecological/life history traits and environmental conditions demonstrate how multi-foci management is a necessary precursor to any successful conservation action–there is no magic bullet to prevent extinctions. Conservation efforts need to be coupled to substantial increases in international research on the long-term monitoring of amphibian populations [28], [29] and collection of life history and ecological data to effectively mitigate the current meltdown of amphibian biodiversity.

## Materials and Methods

The choice of ecological, life history and environmental variables used in the our analysis was a function of (1) data availability for the largest number of species to maximize sample sizes; (2) identification of the most logical variables shown or believed to be responsible for altering the probability of threat and decline risk in a large number of taxonomically specific species [e.g.], [8,18]; and (3) parsimony considerations to limit the number of models and variable combinations that facilitate interpretation. We were careful to limit our hypotheses to specific combinations of variables under thematic categories so that weak and possible confounded variable combinations could be avoided. We also were particularly mindful of biases that have already been revealed in previous studies linking climate change, disease outbreak, and other factors that affect risk for amphibians.

Data were compiled from various sources including the extensive Global Amphibian Assessment database (GAA) [29] and numerous field guides and expert opinions (see Supporting Information). We adapted the earlier (2005) version of the GAA amphibian species list, but did not include most recently described species (from 2005 onward). Nomenclature and taxonomy follow the current version of the GAA. We did not include introduced taxa outside of their natural geographic ranges. Geographic distribution maps for 5,813 of the 5,918 described amphibian species used for our analyses were assembled and supplied by the GAA.

We compiled ecological, life history and environmental data from a total of 5,717 amphibian species from 3 orders, 48 families and 460 genera (Supplementary Table S1). Of these, 1,801 (46%) were classed as threatened according to IUCN criteria. For the decline-risk analysis (see main text and below), we obtained data on population trends between 1980 and 2004 for 4,027 of the 5,717 species (70%). Of these, 2,454 species (61%) were considered to have declined, 1,545 species (38%) had remained stable, and 28 species (1%) had increased. Due to missing data in some categories (e.g., body size), the final number of species analysed varied according to the model set under consideration. Supplementary Table S1 provides the ranges of species sample sizes used in the analyses (see Results tables for exact numbers).

Information on country distribution, total range area (km^2^), population trend, and habitat distribution of species were obtained exclusively from the GAA, and conservation status was based on applicable versions of the IUCN Red List (www.iucnredlist.org). Reproduction, habits, body size (snout-vent length for anurans, total length for caudates and caecilians), and altitudinal distribution were obtained from field guides, herpetology textbooks, monographs, journal articles, and online amphibian databases and websites (see Supplementary Notes S1); we also included unpublished field data for a few species (see Acknowledgements). In the absence of data (or sources that we could not access) for a particular species, we sometimes assumed similar values based on within-genus trends and information available from closely allied taxa (if experts agreed). We also located original information sources (i.e., original description of types or taxonomic monographs) when popular references provided inconsistent data. Body sizes were based on median values of five categories established for each amphibian order (see Supplementary Table S2). After initial data compilation, we found only three errors (all missing data) in over 1,500 random data field confirmations (error rate = 0.002%), and after corrections, we found no other errors in another 100 random field confirmations in a dataset that included more than 1.25 million values.

We used geographic distribution maps for 5,813 (98.2 %) of 5,918 described amphibian species, assembled and supplied by the GAA [29] for our analyses. Each species' extent-of-occurrence map is a single minimum convex polygon that connects known locations, but includes multiple polygons when there is clear range discontinuity. We extracted human impact and bioclimatic variables of individual species by overlaying each species' distribution map with available data using the Spatial Analyst extension of ArcGIS v9.0. Mean human population density (people·km^−2^) within the geographic range of each species was estimated using the Gridded Population of the World for 1995 at 2.5 arc-minute resolution [30]. This database is derived from human population census data for ca. 127,000 sub-national geographic units based on national population estimates that have been adjusted to match the UN national estimated population for each country.

Extent of habitat loss due to anthropogenic impact was evaluated by using a modified version 3 of the Global Land Cover 2000 dataset (GLC 2000) [31] to calculate percent area converted within each species' geographic range. The GLC 2000 is a compilation of continental land cover maps that categorizes land cover at 1-km^2^ resolution for all land masses except Antarctica. Percent area converted was calculated as percentage of terrestrial area classified as cultivated or managed areas, cropland mosaics, and artificial surfaces and associated areas, in the modified GLC. Following Hoekstra et al. [32], we assumed that past area conversion within each species range was zero.

Mean bioclimatic variables within each species distribution range were estimated using ‘WorldClim’, a global climate database with high spatial resolution (Version 1.4; www.worldclim.org [33]). Climate layers were produced through interpolation of average monthly climate data (i.e., monthly precipitation, and monthly mean, minimum and maximum temperature) from weather stations on a 30 arc-second resolution grid (commonly referred to as “1-km^2^” resolution; ∼0.86 km^2 ^at the equator). The ‘WorldClim’ database was assembled using major climate databases, including Global Historical Climatology Network (GHCN), Food and Agriculture Organization of the United Nations (FAO), World Meteorological Organization (WMO), International Centre for Tropical Agriculture (CIAT), R-Hydronet, among others, and were limited to records from 1950–2000. Climate surfaces were developed using a thin plate smoothing spline algorithm implemented in ANUSPLIN software–a program for interpolating noisy multivariate data with latitude, longitude, and elevation as independent variables.

Compared to other widely used global climate databases (e.g., see New et al. [34]), the ‘WorldClim’ database has a number of advantages for analysing taxa with small and restricted geographic ranges such as amphibians: (1) bioclimatic data have high spatial resolution; (2) a large number of weather station records are used; (3) it uses improved elevation data; and (4) a greater degree of knowledge on spatial patterns of uncertainty in data are incorporated. The six aggregated bioclimatic variables we selected for analysis are biologically relevant, representing annual trends and limiting environmental factors derived from monthly temperature (mean, minimum and maximum) and rainfall values. These variables include mean annual mean temperature (in ° C), maximum temperature of warmest month, minimum temperature of coldest month, annual precipitation (in mm), precipitation of wettest month, and precipitation of driest month, estimated within each species geographic range. As observed by Cooper et al. [8], we believe that data quality issues between range map (area of occupancy) vs. GAA geographical range map are minimal.

## Analysis

To avoid potentially spurious or statistically intractable problems common in large-scale correlative studies, our model-building strategy used existing knowledge from other studies [2], [4]–[6] and logic to construct a plausible set of *a priori* hypotheses regarding the relationship between threat risk its putative drivers. This design avoided an all-subsets approach by testing specific hypothesis (rather than all possible term combinations) which essentially amounts to model data-mining. We split the modelling approach into two phases to avoid over-parameterizing models: (1) Phase 1 examined the relationship between threat risk and life history correlates *body size*, *geographic range*, life history *habit*, *spawn site*, *reproductive cycle*, *reproductive mode*, presence/absence of *parental care* and *fertilization type*. The terms *body size* and *geographic range* were log-transformed, and all other variables were coded as categorical factors. Various combinations of life history terms were built under life history themes (*n = *33 models; Supplementary Table S3), and we also considered 7 interaction terms (Table S3) combined with the single-term saturated model (Table S3). We also examined the evidence for nonlinear (quadratic) relationships between the threat risk and *geographic range* based on recent findings [8]. (2) Phase 2 incorporated terms from the most parsimonious models (model ranking described below) supported in Phase 1, with addition of environmental terms *mean ambient temperature*, *annual temperature seasonality*, *mean annual precipitation*, *annual precipitation seasonality*, *human density* and *proportional habitat loss*. Terms were combined under themes as in Phase 1, with 4 interactions considered (*n = *74 models; Supplementary Table S4). *Annual temperature seasonality* and *annual precipitation seasonality* were calculated as the square root of the difference between mean annual maximum and minimum values. *Proportional habitat loss* was arcsine-square root transformed to normalize its distribution. Nonlinear (quadratic) relationships between the response variable and *mean ambient temperature* and *mean annual precipitation* [8] were considered. Given that the processes driving population decline are often decoupled from those ultimately determining extinction [17], [21], we hypothesized that a different set of correlates might apply to the probability of population decline. The entire two-phase process was therefore repeated for the response *decline risk*–whether or not there was evidence for population decline for each species

Each hypothetical relationship was fitted as a specific generalized linear mixed-effect model (GLMM) relating the response variables (threat risk, decline risk) using the lmer function of the lme4 library in the *R* Package [35]. Threat risk (i.e., IUCN Red Listed or not) was coded as a binomial response variable and each trait as a linear predictor (fixed effects), assigning each model a binomial error distribution and a logit link function. Decline risk was coded similarly, with species showing no evidence of decline coded as ‘no decline’.

Species are phylogenetic units with shared evolutionary histories and are therefore not statistically independent units [36]. Indeed, previous work has demonstrated that the risk of decline and/or extinction may vary among families in amphibians [7]. It was therefore necessary to decompose variance across species by coding the random-effects error structure of GLMM as a hierarchical taxonomic (Order/Family) effect (adjusting the random effect's intercept term) [37], [38]. We had insufficient replication within some families to include genus in the nested random effect (GLMMs failed to converge), but we expect that even with sufficient replication of genera there would be little effect on model goodness-of-fit given the small contribution of phylogenetic control revealed by contrasting GLMMs with GLMs (function glm in the *R* Package) (see Results). Therefore, we are confident that our level of taxonomic control is sufficient to account for the majority of phylogenetic relatedness.

GLMMs are more appropriate than the independent-contrasts approach [36] in situations where a complete phylogeny of the study taxon is unavailable, when categorical variables are included in the analysis, and when model selection, rather than hypothesis testing, is the statistical paradigm used. The amount of variance in the threat probability response variable captured by each combination of terms considered (see below) was assessed as the percent deviance explained (%DE), which is a measure of a model's goodness-of-fit to the data [39].

In addition to accounting for phylogenetic relatedness in our mixed-effects models, we controlled statistically for potential spatial autocorrelation among the species examined (see Supplementary Tables S9–10). When one species' fate is correlated with that of its neighbours beyond that accounted for by environmental variables (such as the ones cited above), then spurious relationships may arise [e.g., 8]. Previous attempts to control for phylogenetic relatedness and spatial autocorrelation have been problematic given a lack of targeted statistical development; however, the mixed-effects linear modelling approach offers some advantages here. We first calculated the great-circle distance matrix of the pair-wise geographic distances (in miles to account for a spheroid) between the species' range centroids using the rdist.earth function of the fields library in the *R* Package. We then calculated the mean geographic distance for each species based on this matrix and incorporated this covariate as a random-effects slope within the random term of the models (e.g., “distance|Order/Family” in the format required for lmer objects in the *R* language [38])

We used an index of Kullback-Leibler (K-L) information loss to assign relative strengths of evidence to the different competing models in each model set–Akaike's information criterion corrected for small sample sizes (AIC*_c_*), as well as the dimension-consistent Bayesian Information Criterion (BIC) [39]. These indices of model parsimony identify relative evidence of model(s) from a set of candidate models. Relative likelihoods of candidate models were calculated using AIC*_c_* and BIC weights [39], with weight (*w*AIC*_c_* and *w*BIC) of any particular model varying from 0 (no support) to 1 (complete support) relative to the entire model set. However, the K-L prior used to justify AIC*_c_* weighting can favour more complex models when sample sizes are large [37], [39] (as was the case for our dataset), so we considered BIC weighting to determine the contribution of the most important correlates of extinction risk (essentially, posterior model probabilities given an uninformative prior) [37]), and AIC*_c_* weighting to identity tapering effects [37], [39]. Sample size for each model was reduced in most cases due to some missing data in some of the hypothesized correlates (updated sample sizes given in Results).

## Supporting Information

Table S1 (0.03 MB DOC)

Table S2 (0.07 MB DOC)

Table S3 (0.06 MB DOC)

Table S4 (0.06 MB DOC)

Table S5 (0.06 MB DOC)

Table S6 (0.05 MB DOC)

Table S7 (0.05 MB DOC)

Table S8 (0.05 MB DOC)

Table S9 (0.08 MB DOC)

Table S10 (0.05 MB DOC)

Supplementary Notes S1 (0.05 MB DOC)
