# Supplementary material for: Measuring the Meltdown: Drivers of Global Amphibian Extinction and Decline
Source: PLoS One. 2008 Feb 20;3(2):e1636. doi: 10.1371/journal.pone.0001636 (PMC2238793; doi:10.1371/journal.pone.0001636)
Supplement: Table S1 — (0.03 MB DOC) [file pone.0001636.s001.doc]

Supporting Table S1. Summary of amphibian species’ taxonomic sample distribution. The final number of species analysed depended on the particular set of attributes included in the model sets (Tables 1-2, S4-SX), so ranges are provided here.

| **Order** | **Families** | Genera | **Species** | **Analyzed sample size** |
| --- | --- | --- | --- | --- |
| Anura | 33 | 365 | 5041 | 2267 – 2746 |
| Caudata | 10 | 62 | 507 | 211 – 303 |
| Gymnophiona | 5 | 33 | 171 | 3 – 16 |
|  |  |  |  |  |
| TOTALS | 48 | 460 | 5717 | 2494 – 3052 |
