# Supplementary material for: Measuring the Meltdown: Drivers of Global Amphibian Extinction and Decline
Source: PLoS One. 2008 Feb 20;3(2):e1636. doi: 10.1371/journal.pone.0001636 (PMC2238793; doi:10.1371/journal.pone.0001636)
Supplement: Table S2 — (0.07 MB DOC) [file pone.0001636.s002.doc]

**Supporting Table S2.** Summary of amphibian species’ ecological, life history and environmental attributes with a list of the species frequency (available data) for the different category levels.

| **Parameter**  **Abbreviation** | **Description** | **Levels** | **Number of Species**  **with data** |
| --- | --- | --- | --- |
| BS | *body size* (length) | continuous (mm) | 4514 |
|  |  |  |  |
| RG | geographic *range* | continuous (km^2^) | 5633 |
|  |  |  |  |
| HB | life history *habit* | aquatic | 288 |
|  |  | arboreal | 681 |
|  |  | terrestrial | 3056 |
|  |  | aquatic & terrestrial | 1615 |
|  |  |  |  |
| SS | *spawn site* | aquatic & terrestrial | 76 |
|  |  | arboreal/phytotelms | 511 |
|  |  | terrestrial | 1900 |
|  |  | parent | 93 |
|  |  | aquatic | 2898 |
|  |  |  |  |
| RC | *reproductive cycle* | aseasonal | 639 |
|  |  | seasonal | 4866 |
|  |  |  |  |
| RM | *reproductive mode* | oviparous | 3868 |
|  |  | viviparous & ovoviviparous | 68 |
|  |  | direct development | 1670 |
|  |  |  |  |
| PC | *parental care* | absent | 3402 |
|  |  | present | 2176 |
|  |  |  |  |
| FT | *fertilization* | external | 5079 |
|  |  | internal | 617 |
|  |  |  |  |
| TM | *mean temperature* | continuous (ºC) | 5629 |
|  |  |  |  |
| TV | *temperature seasonality* | continuous (ºC) | 5629 |
|  |  |  |  |
| PM | *mean precipitation* | continuous (mm) | 5629 |
|  |  |  |  |
| PV | *precipitation seasonality* | continuous (mm) | 5629 |
|  |  |  |  |
| HD | *human density* | continuous (people·km^2^) | 5555 |
|  |  |  |  |
| HL | *habitat loss* | continuous (% lost) | 5640 |
