# Supplementary material for: Measuring the Meltdown: Drivers of Global Amphibian Extinction and Decline
Source: PLoS One. 2008 Feb 20;3(2):e1636. doi: 10.1371/journal.pone.0001636 (PMC2238793; doi:10.1371/journal.pone.0001636)
Supplement: Table S3 — (0.06 MB DOC) [file pone.0001636.s003.doc]

Supporting Table S3. Ecological and life history models. Generalized linear mixed-effects models used to examine the correlation between amphibian ecological and life history attributes and threat/decline risk. Model combinations, derived *a priori*, represent particular analytical ‘themes’ grouping related traits. Terms include BS = *body size*, RG = *range* (km^2^), HB = life history *habit*, SS = *spawning site*, RC = *reproductive cycle*, RM = *reproductive mode*, PC = *presence/absence of parental care*, and FT = *fertilization type*.

| Model  No. | Model | Analytical Theme |
| --- | --- | --- |
| 1 | ~BS | allometry |
| Habitat models | |  |
| 2 | ~RG | range |
| 3 | ~RG+RG^2^ | non-linear range (quadratic) |
| 4 | ~BS+RG | allometry+range |
| 5 | ~BS+RG+RG^2^ | allometry+non-linear range |
| 6 | ~BS+RG+HB | allometry+range+habit |
| 7 | ~BS+RG+HB+SS | allometry+range+habit+spawn site |
| 8 | ~BS+HB+SS | allometry+habit+spawn site |
| 9 | ~BS+HB | allometry+habitat |
| 10 | ~BS+SS | allometry+spawn site |
| Reproductive models | |  |
| 11 | ~BS+RC | allometry+reproductive cycle |
| 12 | ~BS+RM | allometry+reproductive mode |
| 13 | ~BS+RC+RM | allometry+reproductive cycle/mode |
| 14 | ~BS+RC+RM+PC | allometry+reproductive cycle/mode+care |
| 15 | ~BS+RM+PC | allometry+reproductive mode+care |
| 16 | ~BS+RC+PC | allometry+reproductive cycle+care |
| 17 | ~BS+FT | allometry+fertilization type |
| 18 | ~BS+FT+PC | allometry+fertilization+care |
| 19 | ~BS+PC | allometry+parental care |
| Combination models | |  |
| 20-30 | combined habitat & reproductive models | - |
| 31 | ~BS+RG+HB+SS+RC+RM+PC+FT | saturated (all predictors included) |
| 32 | ~BS+RG+HB+SS+RC+RM+PC+FT+RG*BS | saturated+range*body size |
| 33 | ~BS+RG+HB+SS+RC+RM+PC+FT+HB*RG | saturated+habit*range |
| 34 | ~BS+RG+HB+SS+RC+RM+PC+FT+BS*HB | saturated+body size*habit |
| 35 | ~BS+RG+HB+SS+RC+RM+PC+FT+RG*RC | saturated+range*reprod cycle |
| 36 | ~BS+RG+HB+SS+RC+RM+PC+FT+RG*RM | saturated+range*reprod mode |
| 37 | ~BS+RG+HB+SS+RC+RM+PC+FT+BS*RC | saturated+body size*reprod cycle |
| 38 | ~BS+RG+HB+SS+RC+RM+PC+FT+BS*RM | saturated+body size*reprod mode |
| 39 | fully saturated with all interactions together | - |
| 40 | ~1 (single mean value for all species) | null (no predictors of extinction risk) |
