# Supplementary material for: Measuring the Meltdown: Drivers of Global Amphibian Extinction and Decline
Source: PLoS One. 2008 Feb 20;3(2):e1636. doi: 10.1371/journal.pone.0001636 (PMC2238793; doi:10.1371/journal.pone.0001636)
Supplement: Table S5 — (0.06 MB DOC) [file pone.0001636.s005.doc]

Supporting Table S5. Environmental context models. Generalized linear mixed-effects models used to examine the correlation between environmental context and amphibian decline risk, after controlling for life history differences not due to phylogeny (see Supplementary Table S3). Model combinations, derived *a priori*, represent particular analytical ‘themes’ grouping related environmental traits. All terms contain the life history traits of *range* (RG), *range*^2^ (quadratic) and *body size* (BS) as determined from Phase 1 of the analysis (Table 2). Additional terms include TM = *mean ambient temperature*, TV = *annual temperature seasonality*, PM = *mean annual precipitation*, PV = *annual precipitation seasonality*, HD = *human density*, and HL = *proportional habitat loss*.

| Model  No. | Model | Analytical Theme |
| --- | --- | --- |
| 1 | ~BS+RG+RG^2^ | life history control (best model from Results) |
| Temperature & precipitation models | |  |
| 2 | ~BS+RG+RG^2^+TM | temp mean |
| 3 | ~BS+RG+RG^2^+TM+TM^2^ | quadratic temp |
| 4 | ~BS+RG+RG^2^+PM | precip mean |
| 5 | ~BS+RG+RG^2^+PM+PM^2^ | quadratic precip |
| 6 | ~BS+RG+RG^2^+TV | temp seasonality |
| 7 | ~BS+RG+RG^2^+PV | precip seasonality |
| 8 | ~BS+RG+RG^2^+TM+TV | temp mean & seasonality |
| 9 | ~BS+RG+RG^2^+PM+PV | precip mean & seasonality |
| 10 | ~BS+RG+RG^2^+TM+PM | temp & precip mean |
| 11 | ~BS+RG+RG^2^+TM+PV | temp mean & precip seasonality |
| 12 | ~BS+RG+RG^2^+TV+PM | temp seasonality & precip |
| 13 | ~BS+RG+RG^2^+TV+PV | temp & precip seasonality |
| 14 | ~BS+RG+RG^2^+TM+TV+PV | temp mean & seasonality; precip seasonality |
| 15 | ~BS+RG+RG^2^+TM+TV+PM | temp mean & seasonality; precip mean |
| 16 | ~BS+RG+RG^2^+TM+PM+PV | temp & precip mean; precip seasonality |
| 17 | ~BS+RG+RG^2^+TV+PM+PV | temp & precip seasonality; precip mean |
| 18 | ~BS+RG+RG^2^+TM+TV+PM+PV | temp & precip seasonality & mean |
| 19 | ~BS+RG+RG^2^+PM+TM+TM^2^ | quadratic temp; precip mean |
| 20 | ~BS+RG+RG^2^+TM+PM+PM^2^ | quadratic precip; temp mean |
| 21 | ~BS+RG+RG^2^+PV+TM+TM^2^ | quadratic temp; precip seasonality |
| 22 | ~BS+RG+RG^2^+TM+TM^2^+PM+PM^2^ | quadratic temp; quadratic precip |
| 23 | ~BS+RG+RG^2^+TM+TM^2^+TV+PM | quadratic temp; temp seasonality; precip mean |
| Human disturbance models | |  |
| 24 | ~BS+RG+RG^2^+HD | human density |
| 25 | ~BS+RG+RG^2^+HL | habitat loss |
| 26 | ~BS+RG+RG^2^+HD+HL | human density+habitat loss |
| Combination models | |  |
| 27-68 | Combined temp & human disturbance models | - |
| 69 | ~BS+RG+RG^2^+TM+TV+PM+PV+HD+HL | saturated (all predictors included) |
| 70 | ~BS+RG+RG^2^+TM+TV+PM+PV+HD+HL+RG*TV | saturated+range*temp seasonality |
| 71 | ~BS+RG+RG^2^+TM+TV+PM+PV+HD+HL+RG*PV | saturated+range*precip seasonality |
| 72 | ~BS+RG+RG^2^+TM+TV+PM+PV+HD+HL+RG*HD | saturated+range*human density |
| 73 | ~BS+RG+RG^2^+TM+TV+PM+PV+HD+HL+RG*HL | saturated+range*habitat loss |
| 74 | fully saturated with all interactions together | - |
| 75 | ~1 (single mean value for all species) | null (no predictors of extinction risk) |
