# Supplementary material for: Measuring the Meltdown: Drivers of Global Amphibian Extinction and Decline
Source: PLoS One. 2008 Feb 20;3(2):e1636. doi: 10.1371/journal.pone.0001636 (PMC2238793; doi:10.1371/journal.pone.0001636)
Supplement: Table S6 — (0.05 MB DOC) [file pone.0001636.s006.doc]

Table S6. Correlates of amphibian threat risk (full dataset; without spatial autocorrelation). The five most parsimonious generalized linear mixed-effects models investigating (a) life history correlates of threat risk (*n* = 3,365) and (b) environmental context, after accounting for effects of range and body size (*n* = 3,474). Models include nested (hierarchical) taxonomic (Order/Family) random intercepts and geographic distance random slopes to account for spatial autocorrelation. Models were ranked according to the Bayesian Information Criterion (BIC). For ecology/life history models, the five most highly BIC-ranked models accounted for > 99 % of the posterior model weight (*w*BIC) of the total of 40 models considered. For environmental context, model weights were more evenly distributed among the 5 most highly ranked of the 75 models considered. Terms shown are RG = *range* (km^2^), BS = *body size*, TM = *mean temperature*, PV = *precipitation range*, PM = *mean precipitation*, TV = *temperature range*, HL = *% habitat lost*, HD = *human density* (people/km^2^). Also shown are number of parameters (*k*), maximised log-likelihood (*LL*), difference in BIC for each model from the most parsimonious model (ΔBIC ), model weight (*w*BIC), percent deviance explained (%DE) in the response variable (threat probability) by the model under consideration, and the difference between the %DE for the current environmental context model and the base ~BS+RG+RG^2^ model (Δ%DE).

| Model | *k* | *LL* | ΔBIC | *w*BIC | %DE | %DE |
| --- | --- | --- | --- | --- | --- | --- |
|  |  |  |  |  |  |  |
| (a) Ecology/life-history |  |  |  |  |  |  |
| RG+RG^2^ | 6 | -1096.068 | 0.000 | 0.623 | 48.56 |  |
| BS+RG+RG^2^ | 7 | -1093.667 | 1.006 | 0.377 | 48.67 |  |
| RG | 5 | -1116.606 | 35.339 | <0.001 | 47.60 |  |
| BS+RG | 6 | -1114.299 | 36.552 | <0.001 | 47.70 |  |
| saturated; no interactions | 19 | -1078.529 | 40.351 | <0.001 | 49.38 |  |
|  |  |  |  |  |  |  |
| (b) Environmental context |  |  |  |  |  |  |
| BS+RG+RG^2^+TM+TV+PV | 10 | -1086.161 | 0.000 | 0.644 | 50.47 | 1.80 |
| BS+RG+RG^2^+TV+PV | 9 | -1090.810 | 3.531 | 0.110 | 50.25 | 1.58 |
| BS+RG+RG^2^+TM+TV+PV+HL | 11 | -1085.235 | 3.948 | 0.089 | 50.51 | 1.84 |
| BS+RG+RG^2^+TM+TV+PV+HD | 11 | -1085.662 | 4.810 | 0.058 | 50.49 | 1.82 |
| BS+RG+RG^2^+TM+PV | 9 | -1091.462 | 4.847 | 0.057 | 50.22 | 1.55 |
