# Supplementary material for: Measuring the Meltdown: Drivers of Global Amphibian Extinction and Decline
Source: PLoS One. 2008 Feb 20;3(2):e1636. doi: 10.1371/journal.pone.0001636 (PMC2238793; doi:10.1371/journal.pone.0001636)
Supplement: Table S7 — (0.05 MB DOC) [file pone.0001636.s007.doc]

Table S7. Correlates of amphibian threat risk (full dataset; with spatial autocorrelation). The five most parsimonious generalized linear mixed-effects models investigating (a) life history correlates of threat risk (*n* = 3,365) and (b) environmental context, after accounting for effects of range and body size (*n* = 3,474). Models include nested (hierarchical) taxonomic (Order/Family) random intercepts and geographic distance random slopes to account for spatial autocorrelation. Models were ranked according to the Bayesian Information Criterion (BIC). For ecology/life history models, the five most highly BIC-ranked models accounted for > 99 % of the posterior model weight (*w*BIC) of the total of 40 models considered. For environmental context, model weights were more evenly distributed among the 5 most highly ranked of the 75 models considered. Terms shown are RG = *range* (km^2^), BS = *body size*, TM = *mean temperature*, PV = *precipitation range*, PM = *mean precipitation*, TV = *temperature range*, HL = *% habitat lost*, HD = *human density* (people/km^2^) Also shown are number of parameters (*k*), maximised log-likelihood (*LL*), difference in BIC for each model from the most parsimonious model (ΔBIC ), model weight (*w*BIC), percent deviance explained (%DE) in the response variable (threat probability) by the model under consideration, difference in BIC for each model from the most parsimonious model (ΔBIC ), and the difference between the %DE for the current environmental context model and the base ~BS+RG+RG^2^ model (Δ%DE).

| Model | *k* | *LL* | ΔBIC | *w*BIC | %DE | %DE |
| --- | --- | --- | --- | --- | --- | --- |
|  |  |  |  |  |  |  |
| (a) Ecology/life-history |  |  |  |  |  |  |
| BS+RG+RG^2^ | 9 | -1091.980 | 0.000 | 0.587 | 48.75 |  |
| RG+RG^2^ | 8 | -1095.218 | 0.702 | 0.413 | 48.60 |  |
| BS+RG | 8 | -1112.686 | 35.736 | <0.001 | 47.78 |  |
| RG | 7 | -1115.920 | 36.390 | <0.001 | 47.63 |  |
| saturated; no interactions | 21 | -1076.184 | 45.726 | <0.001 | 49.50 |  |
|  |  |  |  |  |  |  |
| (b) Environmental context |  |  |  |  |  |  |
| BS+RG+RG^2^+TM+PM+PV | 12 | -1086.163 | 0.000 | 0.363 | 50.45 | 1.70 |
| BS+RG+RG^2^+PM+PV | 11 | -1089.617 | 1.085 | 0.211 | 50.29 | 1.54 |
| BS+RG+RG^2^+TM+TV+PV | 12 | -1087.181 | 1.908 | 0.140 | 50.40 | 1.65 |
| BS+RG+RG^2^+TV+PV | 11 | -1091.013 | 3.906 | 0.051 | 50.23 | 1.48 |
| BS+RG+RG^2^+TM+TV+PM+PV+HL | 14 | -1082.719 | 4.621 | 0.036 | 50.61 | 1.86 |
