# Supplementary material for: Measuring the Meltdown: Drivers of Global Amphibian Extinction and Decline
Source: PLoS One. 2008 Feb 20;3(2):e1636. doi: 10.1371/journal.pone.0001636 (PMC2238793; doi:10.1371/journal.pone.0001636)
Supplement: Table S8 — (0.05 MB DOC) [file pone.0001636.s008.doc]

Table S8. Correlates of amphibian threat risk (reduced dataset; without spatial autocorrelation). The five most parsimonious generalized linear mixed-effects models investigating (a) life history correlates of threat risk (*n* = 2,494) and (b) environmental context, after accounting for effects of range and body size (*n* = 2,584). Models include nested (hierarchical) taxonomic (Order/Family) random intercepts and geographic distance random slopes to account for spatial autocorrelation. Models were ranked according to the Bayesian Information Criterion (BIC). For ecology/life history models, the five most highly BIC-ranked models accounted for > 99 % of the posterior model weight (*w*BIC) of the total of 40 models considered. For environmental context, model weights were more evenly distributed among the 5 most highly ranked of the 75 models considered. Terms shown are RG = *range* (km^2^), BS = *body size*, HB = *habit*, TM = *mean temperature*, PV = *precipitation range*, PM = *mean precipitation*, TV = *temperature range*, HL = *% habitat lost*, HD = *human density* (people/km^2^) Also shown are number of parameters (*k*), maximised log-likelihood (*LL*), difference in BIC for each model from the most parsimonious model (ΔBIC ), model weight (*w*BIC), percent deviance explained (%DE) in the response variable (threat probability) by the model under consideration, difference in BIC for each model from the most parsimonious model (ΔBIC ), and the difference between the %DE for the current environmental context model and the base ~BS+RG model (Δ%DE).

| Model | *k* | *LL* | ΔBIC | *w*BIC | %DE | %DE |
| --- | --- | --- | --- | --- | --- | --- |
|  |  |  |  |  |  |  |
| (a) Ecology/life-history |  |  |  |  |  |  |
| BS+RG | 6 | -583.276 | 0.000 | 0.685 | 46.08 |  |
| RG | 5 | -587.178 | 2.719 | 0.176 | 45.72 |  |
| BS+RG+RG^2^ | 7 | -582.603 | 3.814 | 0.102 | 46.14 |  |
| RG+RG^2^ | 6 | -586.521 | 6.562 | 0.026 | 45.78 |  |
| BS+RG+HB | 9 | -580.534 | 9.829 | 0.005 | 46.34 |  |
|  |  |  |  |  |  |  |
| (b) Environmental context |  |  |  |  |  |  |
| BS+RG+TM+PV | 8 | -573.809 | 0.000 | 0.543 | 48.76 | 2.68 |
| BS+RG+TM+TV+PV | 9 | -571.942 | 1.322 | 0.281 | 48.93 | 2.85 |
| BS+RG+TM+PV+HL | 9 | -573.488 | 4.449 | 0.059 | 48.79 | 2.71 |
| RG+BS+TM+PV+HD | 9 | -573.781 | 5.043 | 0.044 | 48.76 | 2.68 |
| RG+BS+TM+TV+PV+HL | 10 | -571.657 | 5.841 | 0.029 | 48.95 | 2.87 |
