# Supplementary material for: Measuring the Meltdown: Drivers of Global Amphibian Extinction and Decline
Source: PLoS One. 2008 Feb 20;3(2):e1636. doi: 10.1371/journal.pone.0001636 (PMC2238793; doi:10.1371/journal.pone.0001636)
Supplement: Table S10 — (0.05 MB DOC) [file pone.0001636.s010.doc]

Table S10. Correlates of amphibian decline risk (reduced dataset; without spatial autocorrelation). The five most parsimonious generalized linear mixed-effects models investigating (a) life history correlates of decline risk (*n* = 3,045) and (b) environmental context, after accounting for effects of life history correlates (top-ranked ecology/life-history model denoted as ‘lhb’ – life-history base) (*n* = 3,025). Models include nested (hierarchical) taxonomic (Order/Family) random intercepts and geographic distance random slopes to account for spatial autocorrelation. Models were ranked according to the Bayesian Information Criterion (BIC). For ecology/life history models, the five most highly BIC-ranked models accounted for > 99 % of the posterior model weight (*w*BIC) of the total of 40 models considered. For environmental context, model weights were more evenly distributed among the 5 most highly ranked of the 75 models considered. Terms shown are RG = *range* (km^2^), BS = *body size*, HB = *habit* , RC = *reproductive cycle*, RS = *reproductive strategy*, PC = *presence/absence of parental care*, SS = *spawning site* and FT = *fertilization type*, TM = *mean temperature*, PV = *precipitation range*, PM = *mean precipitation*, TV = *temperature range*, HL = *% habitat lost*, HD = *human density* (people/km^2^). Also shown are number of parameters (*k*), maximised log-likelihood (*LL*), difference in BIC for each model from the most parsimonious model (ΔBIC ), model weight (*w*BIC), percent deviance explained (%DE) in the response variable (decline probability) by the model under consideration, and the difference between the %DE for the current environmental context model and the life history base (lhb) model (Δ%DE).

| Model | *k* | *LL* | ΔBIC | *w*BIC | %DE | %DE |
| --- | --- | --- | --- | --- | --- | --- |
|  |  |  |  |  |  |  |
| (a) Ecology/life-history |  |  |  |  |  |  |
| BS+RG+RG^2^+HB+SS+RC+RS+PC+FT | 19 | -1608.657 | 0.000 | 0.997 | 17.02 |  |
| RG+RG^2^ | 7 | -1652.609 | 12.976 | 0.002 | 14.75 |  |
| BS+RG+RG^2^ | 6 | -1655.973 | 13.423 | 0.001 | 14.58 |  |
| BS+RG+HB+RC+RS | 12 | -1652.936 | 45.186 | <0.001 | 14.74 |  |
| BS+RG+HB+RC | 11 | -1657.014 | 47.063 | <0.001 | 14.53 |  |
|  |  |  |  |  |  |  |
| (b) Environmental context |  |  |  |  |  |  |
| lhb…+TM+PV+HL | 22 | -1530.959 | 0.000 | 0.863 | 20.47 | 3.45 |
| lhb…+TM+PV+HL+HD | 23 | -1530.166 | 4.604 | 0.086 | 20.52 | 3.50 |
| lhb…+TM+TV+PV+HL | 23 | -1530.957 | 6.200 | 0.039 | 20.48 | 3.46 |
| lhb…+PM+PV+HL | 21 | -1539.131 | 10.264 | 0.005 | 20.06 | 3.04 |
| lhb…+TM+TV+PV+HL+HD | 24 | -1530.161 | 10.800 | 0.004 | 20.52 | 3.50 |
