# Supplementary material for: Measuring the Meltdown: Drivers of Global Amphibian Extinction and Decline
Source: PLoS One. 2008 Feb 20;3(2):e1636. doi: 10.1371/journal.pone.0001636 (PMC2238793; doi:10.1371/journal.pone.0001636)
Supplement: Supplementary Notes S1 — (0.05 MB DOC) [file pone.0001636.s011.doc]

**Supporting Notes**

The following are the sources of information used to compile the database. Books and expert opinions were used more often than websites. Whenever possible, we asked experts to review the parts of the database they felt qualified to verify.

**Websites**

Global Amphibian Assessment – The World Conservation Union (IUCN), Conservation International (CI), and NatureServe. Global Amphibian Assessment. www.globalamphibians.org (2006). (Accessed: 13 May 2005).

Amphibian Species of the World – Frost, D. R. Amphibian Species of the World: an Online Reference Version 5.0. research.amnh.org/herpetology/amphibia/index.php (American Museum of Natural History, New York, USA, 2007). (Accessed: 19 April 2005).

AmphibiaWeb – [AmphibiaWeb](http://amphibiaweb.org/): Information on amphibian biology and conservation. amphibiaweb.org (Berkeley, California: AmphibiaWeb, 2006). (Accessed: 19 September 2005).

Living Underworld – Miller, J.J. and Livingunderworld.org. (Accessed: 4 January 2006).

Gymnophiona.org – Nelson, N. Gymnophiona.org. gymnophiona.org. (Accessed: 4 June 2006).

Frog Log – Declining Amphibian Populations Task Force. Frog Log. www2.open.ac.uk/biology/froglog (2003). (Accessed: 27 June 2005).

**Books**

Alcala, A. C. & Brown, W. C. *Philippine Amphibians: An Illustrated Field Guide* (Bookmark Press, Makati City, Philippines, 2003).

Arnold, N. *Collins Field Guide to the Reptiles and Amphibians of Britain and Europe* (Harper Collins, London, 2005).

Barker, J., Grigg, G., & Tyler, M. *A Field Guide to Australian Frogs* (Surrey Beatty & Sons, Chipping Norton, 1995).

Bauer, A. M. & Sadlier, R. A. *The Herpetofauna of New Caledonia* (Society for the Study of Amphibians and Reptiles, 2000).

Blair, W. F. *Evolution in the Genus Bufo* (University of Texas Press, Louisiana, 1972).

Berry, P. Y. *The Amphibian Fauna of Peninsula Malaysia* (Tropical Press, Kuala Lumpur, 1975).

Biju, S. D. & Bossuyt, F. New frog family from India reveals an ancient biogeographical link with the Seychelles. *Nature* **425**: 711–714 (2003).

Bourret, R. Les Batraciens de l'Indochine. *Institut Océanographique de l'Indochine* **6**: 1–547 (1942).

Channing, A. *Amphibians of Central and Southern Africa* (Cornell University Press, 2001).

Conant, R., & Collins, J. T. *Peterson Field Guide to Reptiles and Amphibians: Eastern and Central North America* 3^rd^ ed. expanded (Houghton Mifflin, New York, 1998).

Daniel, J. C. *The Book of Indian Reptiles and Amphibians* (Bombay Natural History Society and Oxford Univ. Press, Mumbai, 2002).

Duellman, W. E. *Hylid Frogs of Middle America (Volumes 1 & 2)* (Contributions to Herpetology Series Vol. 18, Society for the Study of Amphibians and Reptiles, 2001).

Duellman, W. E. *Cusco Amazónico:* *The Lives of Amphibians and Reptiles in an Amazonian Rainforest* (Cornell University Press, Ithaca, New York, 2005).

Duellman, W. E. & Trueb, L. S. *Biology of Amphibians* (The Johns Hopkins Univ. Press, Baltimore, 1994).

Dutta, S. K. & Manamendra-Arachchi, K. *The Amphibian Fauna of Sri Lanka* (Wildlife Heritage Trust of Sri Lanka, Colombo, 1996).

Fei, L., Ye, C.-Y., Huang, Y.-A., & Liu, M.-Y. *Atlas of Amphibians of China* (Henan Science and Technical Press, Zhengzhou, China, 1999).

Funk, W. C., Fletcher-Lazo, G., Nogales-Sornosa, F., & Almeida-Reinoso, D. First description of a clutch and nest site for the genus *Caecilia* (Gymnophiona: Caeciliidae). *Herpetological Review* **35**:128–130 (2004).

Gallagher, M. D. *The Amphibians and Reptiles of Bahrain* (Subbuteo Natural History Books, 1971).

Goris, R. C. & Maeda, N. *Guide to the Amphibians and Reptiles of Japan* (Krieger, Florida, 2005).

Guyer, C. & Donnelly, M. A. *Amphibians and Reptiles of La Selva, Costa Rica, and the Caribbean Slope: a comprehensive guide* (California Univ. Press, California, 2005).

Henkel, F.-W. & Schmidt, W. *Amphibians and Reptiles of Madagascar, the Mascarenes, the Seychelles and the Comoros Islands* (Krieger Publishing Company, Florida, 2000).

Inger, R. F. & Stuebing, R. B. *A Field Guide to the Frogs of Borneo* (Borneo Natural History Publishers, Kota Kinabalu, Malaysia, 1997).

Iskandar, D. T. *The Amphibians of Java and Bali* (Research and Development Centre for Biology, The Indonesian Institute of Research, 1998).

Khan, M. S. *Amphibians and Reptiles of Pakistan* (Krieger Publishing Company, Malabar, Florida, 2006).

Lee, J. C. *A Field Guide to the Amphibians & Reptiles of the Maya World: The lowlands of Mexico, northern Guatemala, and Belize* (Cornell Univ. Press, New York, 2000).

Lim, K. P. & Lim, F. L. K. *A Guide to the Amphibians and Reptiles of Singapore* (Singapore Science Centre, Singapore, 1992).

Lynch, J. D. & Duellman, W. E. *Frogs of the Genus Eleutherodactylus in Western Ecuador: Systematics, ecology, and biogeography* (Univ. of Kansas, Natural History Museum, 1997).

Malkmus, R., Manthey, U., Vogel, G., Hoffmann, P. & Kosuch, J. *Amphibians and Reptiles of Mount Kinabalu (North Borneo)* (A.R.G. Gantner Verlag Kommanditgesellschaft, Ruggell, Liechtenstein, 2002).

McCranie, J. R. & Wilson, L. D. *The Amphibians of Honduras (Contributions to Herpetology Series Vol. 19)* (Society for the Study of Amphibians and Reptiles, 2002).

Min, M. S. et al. Discovery of the first Asian plethodontid salamander. *Nature* **435**:87–90 (2005).

Nussbaum, R. A. & Wilkinson, M. On the classification and phylogeny of caecilians (Amphibia: Gymnophiona), a critical review. *Herpetological Monographs* **3**:1–42 (1989).

Petranka, J. W. *Salamanders of the United States and Canada* (Smithsonian Institute Press, Washington, D.C, 1998).

Pough, F. H. et al. *Herpetology* (Prentice Hall, Upper Saddle River, New Jersey, 1998).

Savage, J.M. 2002. *The Amphibians and Reptiles of Costa Rica, a herpetofauna between two continents, between two seas*. University of Chicago Press.

Schleich, H. H. & Kästle, W. *Amphibians and Reptiles of Nepal: Biology, Systematics, Field Guide* (A.R.G. Gantner Verlag, Ruggell, 2002).

Stebbins, R. C. *Peterson Field Guide to Western Reptiles and Amphibians (3^rd^ edition)* (Houghton Mifflin, New York, 2003).

van Kampen, P. N. *The Amphibia of the Indo-Australian Archipelago* (E.J. Brill Ltd., Leiden, 1923).

Walls, J. G. *Poison Dart Frogs of the Family Dendrobatidae: Jewels of the Rainforest* (T.F.H. Publications, New Jersey, 1994).

Zhao, E. M. & Adler, K. *Herpetology of China* (Society for the Study of Amphibians and Reptiles, Lawrence, Kansas, 1993).

Zug, G. R., Vitt, L. J., & Caldwell, J. P. *Herpetology, An Introductory Biology of Amphibians and Reptiles* (Academic Press, San Diego, California, 2001).
